# Older Adults’ Views of Smartphone-Based Monitoring for Dementia Risk

**DOI:** 10.1093/geroni/igaf122.4329

**Published:** 2025-12-31

**Authors:** Katherine Hackett, Heather Wurtz, Frandys Berroa, Hillary Ramos Espinoza, Maria Loizos, Carolyn Zhu, Alex Federman, Mary Sano

**Affiliations:** Icahn School of Medicine at Mount Sinai, New York, New York, United States; University of Southern California, Los Angeles, California, United States; Icahn School of Medicine at Mount Sinai, New York, New York, United States; Icahn School of Medicine at Mount Sinai, New York, New York, United States; Icahn School of Medicine at Mount Sinai, New York, New York, United States; Icahn School of Medicine at Mount Sinai, New York, New York, United States; Icahn School of Medicine at Mount Sinai, New York, New York, United States; Icahn School of Medicine at Mount Sinai, New York, New York, United States

## Abstract

Clinical assessment of function in dementia risk evaluations is challenging and may benefit from passive smartphone monitoring, which enables low-burden, continuous tracking of everyday behaviors outside the clinic. However, acceptability of passive monitoring in older adults is required for such technologies to be useful. To assess acceptability, 17 cognitively normal older adults (mean age=76.9, SD = 8.7; mean education=15.8, SD = 3.4; 59% White, 41% Hispanic) from the Mount Sinai Alzheimer’s Disease Research Center completed a 1-hour semi-structured interview in English or Spanish assessing attitudes toward smartphone monitoring, followed by a 5-point acceptability rating (likelihood of future study participation). Our interview guide drew from the Technology Acceptance Model and preliminary passive smartphone studies to explore a priori themes. Transcripts were analyzed using a hybrid inductive-deductive approach and content analysis. Emergent themes included perceived utility, privacy/ethical concerns, personal characteristics, logistical considerations, and overall comfort level. Key barriers to acceptability included privacy concerns (e.g., unease with monitoring, risk of data breach) which varied by data type (GPS versus call/text meta-data). Some participants questioned the clinical utility and identified potential confounding variables. Factors contributing to acceptability included recognized scope (i.e., perceived value in tracking behaviors not routinely assessed in clinic), altruistic attitudes toward research, clear understanding of privacy protections, and interest in early detection of dementia. Post-interview, 76% were likely to participate in a future digital monitoring study and 94% agreed to recruitment contact. These findings offer insights to enhance acceptability in future clinical implementation, including transparent review of privacy safeguards, existing evidence, and clinical integration logistics.

